# The anti-inflammatory and anti-oxidative effect of a classical hypnotic bromovalerylurea mediated by the activation of NRF2

**DOI:** 10.1093/jb/mvad030

**Published:** 2023-04-11

**Authors:** Haruna Takeda, Yoshihiro Nakajima, Teruaki Yamaguchi, Itaru Watanabe, Shoko Miyoshi, Kodai Nagashio, Hiroki Sekine, Hozumi Motohashi, Hajime Yano, Junya Tanaka

**Affiliations:** Department of Molecular and Cellular Physiology, Graduate School of Medicine, Ehime University, 454, Shitsukawa, Toon, Ehime, 791-0295, Japan; Department of Gene Expression Regulation, Institute of Development, Aging and Cancer, Tohoku University, 4-1, Seiryo-cho, Aoba-ku, Sendai, Miyagi, 980-8575, Japan; Health and Medical Research Institute, National Institute of Advanced Industrial Science and Technology (AIST), 2 217-14, Hayashi-cho, Takamatsu, Kagawa, 761-0301, Japan; Department of Molecular and Cellular Physiology, Graduate School of Medicine, Ehime University, 454, Shitsukawa, Toon, Ehime, 791-0295, Japan; Department of Molecular and Cellular Physiology, Graduate School of Medicine, Ehime University, 454, Shitsukawa, Toon, Ehime, 791-0295, Japan; Department of Molecular and Cellular Physiology, Graduate School of Medicine, Ehime University, 454, Shitsukawa, Toon, Ehime, 791-0295, Japan; Department of Molecular and Cellular Physiology, Graduate School of Medicine, Ehime University, 454, Shitsukawa, Toon, Ehime, 791-0295, Japan; Department of Gene Expression Regulation, Institute of Development, Aging and Cancer, Tohoku University, 4-1, Seiryo-cho, Aoba-ku, Sendai, Miyagi, 980-8575, Japan; Department of Gene Expression Regulation, Institute of Development, Aging and Cancer, Tohoku University, 4-1, Seiryo-cho, Aoba-ku, Sendai, Miyagi, 980-8575, Japan; Department of Molecular and Cellular Physiology, Graduate School of Medicine, Ehime University, 454, Shitsukawa, Toon, Ehime, 791-0295, Japan; Department of Molecular and Cellular Physiology, Graduate School of Medicine, Ehime University, 454, Shitsukawa, Toon, Ehime, 791-0295, Japan

**Keywords:** KEAP1–NRF2, drug action toxins/drugs/xenobiotics, bromovalerylurea, anti-oxidant oxygen, anti-inflammation, Abbreviations: ARE, antioxidant responsive element; BU, bromovalerylurea; CCL2, C-C motif chemokine 2; DMF, dimethyl fumarate; GCLC, glutamate–cysteine ligase catalytic subunit; GCLM, glutamate–cysteine ligase modifier subunit; GSS, glutathione synthetase; GSH, glutathione; Hmox-1, heme oxygenase-1; IL-1β, interluekin-1β; IL-6, interluekin-6; JAK, Janus kinase; KEAP1, Kelch-like ECH-associated protein; NO, nitric oxide; NOS2, NO synthase 2; NRE, NF-κB responsive element; NQO-1, NAD(P)H quinone dehydrogenase; NRF2, nuclear factor erythroid 2-related factor 2/nuclear factor erythroid-derived 2-like 2; TXNRD, thioredoxin–disulfide reductase; ROS, reactive oxygen species

## Abstract

The Kelch-like ECH-associated protein 1–nuclear factor erythroid 2-related factor 2 (KEAP1–NRF2) system plays a central role in redox homeostasis and inflammation control. Oxidative stress or electrophilic compounds promote NRF2 stabilization and transcriptional activity by negatively regulating its inhibitor, KEAP1. We have previously reported that bromovalerylurea (BU), originally developed as a hypnotic, exerts anti-inflammatory effects in various inflammatory disease models. However, the molecular mechanism underlying its effect remains uncertain. Herein, we found that by real-time multicolor luciferase assay using stable luciferase red3 (SLR3) and green-emitting emerald luciferase (ELuc), BU potentiates NRF2-dependent transcription in the human hepatoblastoma cell line HepG2 cells, which lasted for more than 60 h. Further analysis revealed that BU promotes NRF2 accumulation and the transcription of its downstream cytoprotective genes in the HepG2 and the murine microglial cell line BV2. *Keap1* knockdown did not further enhance NRF2 activity, suggesting that BU upregulates NRF2 by targeting KEAP1. Knockdown of *Nfe2l2* in BV2 cells diminished the suppressive effects of BU on the production of pro-inflammatory mediators, like nitric oxide (NO) and its synthase NOS2, indicating the involvement of NRF2 in the anti-inflammatory effects of BU. These data collectively suggest that BU could be repurposed as a novel NRF2 activator to control inflammation and oxidative stress.

## Graphical Abstract

**Graphical Abstract:**
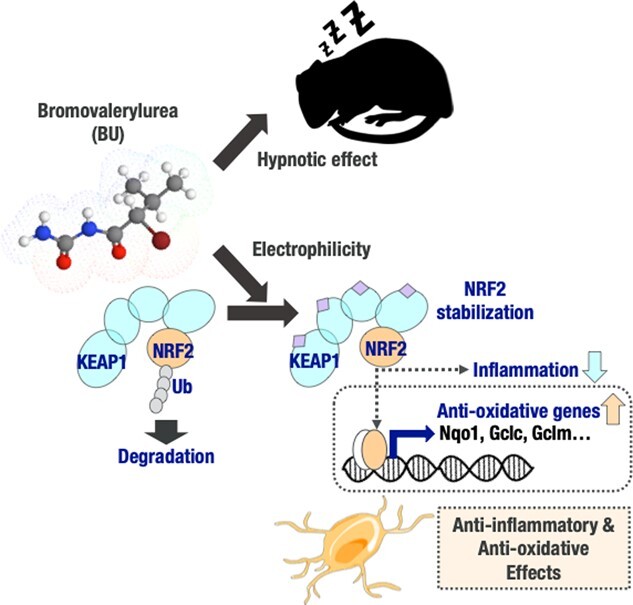

Controlling inflammation remains a major medical challenge, even in this century, and the requirement for new anti-inflammatory drugs is still high *(**1*–*4**)*. Kelch-like ECH-associated protein 1–nuclear factor erythroid 2-related factor 2 (KEAP1–NRF2) system is the central regulator of redox homeostasis *(**5**,**6**)*. The steady-state intracellular level of NRF2 is maintained low by its inhibitory regulator KEAP1. Exposure to oxidative stress or electrophilic compounds inactivates the E3 ligase adapter protein KEAP1, resulting in the stabilization and nuclear translocation of NRF2 *(**7**,**8**)*. Subsequently, NRF2 upregulates its target genes by forming a transcriptional complex on an antioxidant-response element (ARE), combined with small musculoaponeurotic fibrosarcoma (Maf) proteins, other transcriptional regulators and mediators, and exerts cytoprotective functions *(**9*–*11**)*. NRF2 also performs an anti-inflammatory function by repressing the regulation of pro-inflammatory genes *(**12**)*. Therefore, the activation of the KEAP1–NRF2 system leads to anti-oxidative and anti-inflammatory effects, and various activators of this system have been studied for their potential to control inflammation *(**13**,**14**)*. However, only a few compounds have been clinically approved, and some of them, for example, dimethyl fumarate (DMF), have rare but severe adverse effects, such as progressive multifocal leukoencephalopathy *(**15**)*.

Bromovalerylurea (BU) is a classical hypnotic/sedative developed in 1907 *(**16**)* and is still prescribed to patients or is used as an ingredient in over-the-counter antipyretic analgesics, mostly in Asian countries *(**17**,**18**)*. However, BU is currently recognized as a toxicant, because it has been abused and its hypnotic action is weaker than benzodiazepines and other newly developed hypnotics. Therefore, its sale has ceased in most countries *(**19**)*. We have found that BU possesses significant anti-inflammatory and anti-oxidative properties, with therapeutic effects on sepsis *(**20**)*, Parkinson's disease *(**21**)* and secondary neurodegeneration in traumatic brain injury *(**22**)*. However, the molecular mechanism(s) underlying its anti-inflammatory effects are unclear.

Based on its molecular structure, we predicted BU to have an electrophilic moiety (see Fig. 1), suggesting that it could inhibit KEAP1. We found that BU activated NRF2 in both human hepatoblastoma cell line HepG2 and murine microglial cell line BV2. Moreover, the knockdown of NRF2 in BV2 attenuated the anti-inflammatory response of BU. Hence, these data suggest that BU could be repurposed as a novel NRF2 activator to control inflammation and oxidative stress.

## Methods

### Reagents

BU was purchased from FUJIFILM Wako (Osaka, Japan). Lipopolysaccharide (LPS, from *Escherichia coli* serotype 055:B5) and lipoteichoic acid (LTA) were purchased from Sigma-Aldrich, and polyinosinic:polycytidylic acid (poly (I:C)) was purchased from WAKO.

### Cells

HepG2, BV2 cells were cultured in Dulbecco's Modified Eagle Medium (DMEM, FUJIFILM Wako) supplemented with 10% fetal calf serum and an antibiotic cocktail (penicillin–streptomycin-amphotericin B suspension, FUJIFILM Wako). DMEM supplemented with 10 mM HEPES, 0.2 mg/ml BSA (Sigma-Aldrich), 5 mg/ml glucose and 5 μg/ml insulin—5 mM sodium selenite—5 μg/ml transferrin cocktail (Gibco-Thermo Fisher, MA, US) was used as ‘E2 medium’ *(**23**)* to prepare following BV2 samples as described below.

### Constructions and generation of stable cell lines for enhancer assays

Internal control reporter plasmid pTK-ELuc-PEST-R4-Bsd carrying herpes simplex thymidine kinase (TK) promoter and green-emitting ELuc *(**24**)* (Toyobo, Osaka, Japan) from *Pyrearinus termitilluminans (**25**)* was constructed as reported previously *(**26**)*. To monitor stress response pathways, we chose following response element; five tandem repeats of antioxidant response element (ARE, 5′-TCACAGTGACTCAGCAAAATT-3′) for Keap1–Nrf2 pathway, six tandem repeats of nuclear factor-κB (NF-κB) response element (NRE, 5′-CGGAAAGTCCCA-3′) for NF-κB pathway, two tandem repeats of endoplasmic reticulum response element (ERSE, 5′-CCTTCACCAATCGGCGGCCTCCACGACGG-3′) for ER stress pathway, and five tandem repeats of heat shock response element (HSE, 5′-TTCTAGAACGTTCT-3′) for heat shock pathway. Each response element was inserted upstream of TK promoter in a reporter plasmid pTK-SLR3-pENTR *(**27**,**28**)* carrying TK promoter and red-emitting SLR3 (Toyobo) from *Phrixotrix hirtus (**25**,**29**)*. The resulting plasmids were recombined into pϕC31-Neo attB vector *(**30**)* as reported previously *(**28**,**29**)*.

To generate stable cell lines, pTK-ELuc-PEST-R4-Bsd was co-transfected with R4 integrase expression plasmid into HepG2 cells harbouring the multi-integrase mouse artificial chromosome (MI-MAC) vector *(**30**)* (a gift from Dr. M. Oshimura and Dr. Y. Kazuki of Tottori University), and subcultured for selection with 2 μg/ml blasticidin (Thermo Fisher Scientific). The selected cells were further co-transfected with the reporter plasmid carrying response element and ϕC31 integrase expression plasmid, and subcultured for selection with 1 mg/ml G418. Integration of the transgenes into the corresponding sites on the MI-MAC vector was confirmed by genomic PCR.

Reporter gene plasmid vector was also constructed to evaluate the ARE activity in inflammatory microglia cell line BV2. ARE sequence 5′-GACTGAGGGTGACTCAGCAAAATCACTGAGGGTGACTCAGCAAAATCACTGAGGGTGACTCAGCAAAATC-3′ and promoter region of mouse *Nos2* (1500 bp upstream of the transcription start site, prepared by PCR from BV2 genome DNA) were connected upstream of SV40 promoter followed by a firefly luciferase gene and inserted into the pcDNA3 (Invitrogen, MA) vector of which cytomegalovirus promoter is eliminated, respectively. BV2 cell was stably transfected with this construct and selected by G418. BV2 cell that ARE (−) above construct was introduced was also established as the negative control.

### Enhancer assays

HepG2 cells expressing ELuc and SLR3 were seeded in 96-well white clear-bottom plates (Nunc) at a density of 3 x 10^4^ cells/well. After an overnight incubation, the medium was replaced with DMEM without phenol red (Gibco-BRL) supplemented with 10% FBS, 4 mM glutamine, non-essential amino acids (Gibco-BRL), 1 mM pyruvate (FUJIFILM Wako Pure Chemical Corporation), 25 mM HEPES/NaOH (pH 7.0, Nacalai Tesque, Kyoto, Japan) and 300 μM d-luciferin potassium salt (Resem B.V., Lijnden, The Netherlands) with or without BU. Bioluminescence was recorded in real time for 5 s at approximately 30-min intervals in the absence or presence of the R62 long-pass filter (HOYA, Tokyo, Japan) at 37°C in 5% CO_2_ atmosphere under saturated humidity using a microplate-type luminometer (WSL-1565 Kronos HT, ATTO, Tokyo, Japan). ELuc and SLR3 luminescence intensities were calculated as described previously *(**30**)*.

BV2 cells bearing the reporter genes were harvested in PBS and homogenized by ultrasonic wave (UH-50 SMT-company, Tokyo, Japan) following the incubation of BU for 6 h. Luminescence obtained by addition of the luminescence substrate PGL100 reagent (TOYO INK, Tokyo) were measured in FlexStation3 (Molecular Device, CA).

**Fig. 1:**
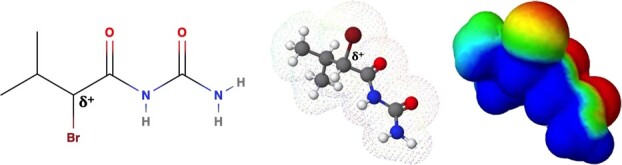
**The molecular structure of BU.** From left to right, the structural formula, the ball-and-stick model, and the pseudo-color presentation of the electron density. Warm color represents strong electrophilicity.

### Transient knockdown experiments

To silence *Keap1* in HepG2 cells, we performed transient knockdown experiment using siRNA. Briefly, siRNAs (10 nM) were transfected into cells using ‘Lipofectamine RNAiMAX. Transfection Reagent (Thermo Fisher Scientific)’ according to the manufacturer's protocol. Keap1 siRNA were purchased from Dharmacon (cat.no. L012453000020). MISSION siRNA Universal Negative Controls (Sigma-Aldrich) were used as controls. 24 h after transfection, culture media were changed. After incubation for another 24 h, cells were treated with or without BU for subsequent assays.

**Fig. 2:**
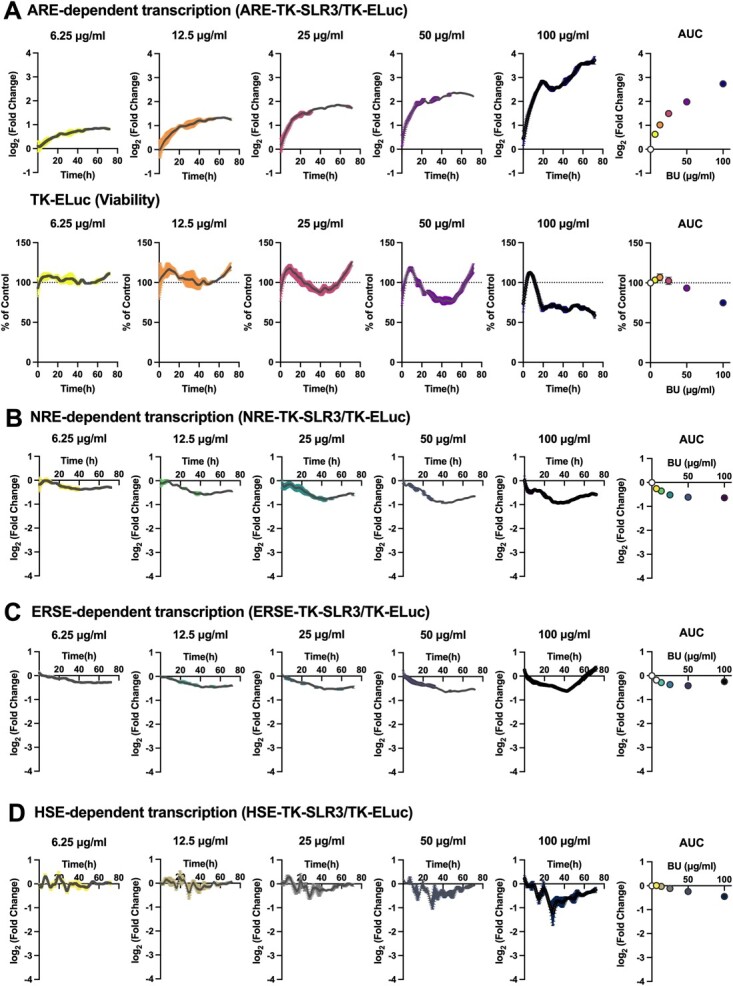
**BU enhances ARE-dependent transcriptional activity in a dose-dependent manner*.*** A. Effects of BU on ARE-dependent transcription (upper panels), which was calculated as log_2_ (ARE-TK-SLR3 (% of control)/TK-ELuc (% of control)). TK-ELuc (% of control) was shown in lower panels. B–D. Effects of BU on NRE-dependent (B), ERSE-dependent (C), HSE-dependent (D) transcription. Dose dependencies are summarized in the areas under the curves (AUC) at the right-most panel. Data are shown as means ± standard deviations (*n* = 3).

### Stable knockdown experiments

pLKO1 puro NRF2 shRNA was purchased from Sigma-Aldrich, and stably transfected to BV2 cell with selection by puromycin. BV2 cell expressing the scrambled sequence shRNA *(**31**)* was also established for the negative control of knockdown.

**Fig. 3:**
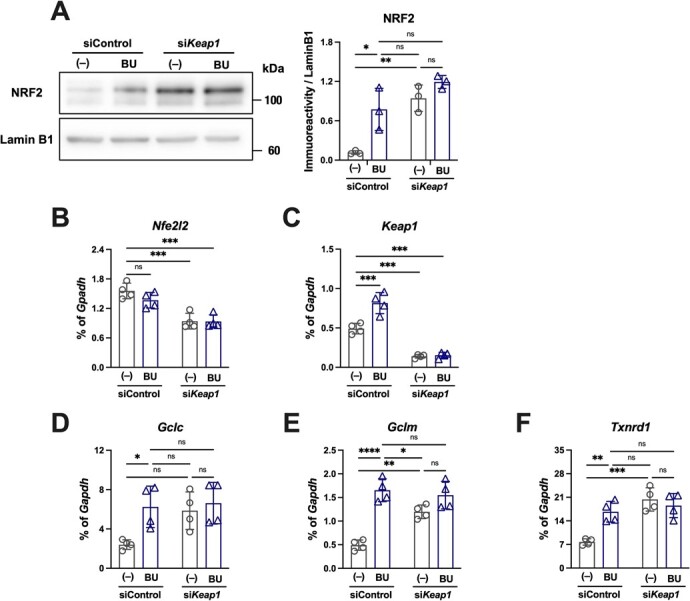
**BU enhances NRF2 accumulation and transcriptional activity by inhibiting its negative regulator, KEAP1.** A–F. HepG2 cells were treated with siRNA against Keap1 and/or BU. Immunoblot analyses of NRF2 protein levels in nuclear fractions (A) and qPCR measurement of the factors related to NRF2 (B, *Nfe2l2*; C, *Keap1*; D, *Gclc*; E, *Gclm*; and F, *Txnrd1*) were executed. The mRNA levels were represented as ratios of *Gapdh* (B–F). ^*^P < 0.05, ^**^P < 0.01, ^***^P < 0.001, and ^****^P < 0.0001 between indicated conditions.

### Immunoblotting

BV2 cells were incubated with BU (100 μg/ml), in the absence or presence of LPS (1 μg/ml) in E2 medium for 3 h (NRF2), 24 h (NOS2), respectively. HepG2 cells were incubated with BU (100ug/ml) in DMEM for 4 h. After incubation, whole cell lysates were prepared using Laemmli sample buffer containing phosphatase inhibitor cocktail solution II (FUJIFILM Wako) *(**32**)*. Nuclear lysates were prepared as described below. The lysates were then subjected to immunoblotting as described elsewhere using antibodies listed in Supplementary Table S1.

### Nuclear extraction for Immunoblotting

For BV2 cells, nuclear extract was prepared using nuclear extraction kit according to the manufacturer's instruction (Active Motif, CA). For HepG2 cells, cells were incubated with hypotonic buffer (10 mM Tris, 15 mM MgCl_2_, 10 mM KCl). After incubation for 10 min, cells were supplemented with 1% NP-40 and vortexed to destroy the cytoplasmic membrane. The pellet was collected as a nuclear fraction and subjected to immunoblotting.

### Quantitative reverse transcriptase PCR

BV2 cells were incubated with or without LPS and BU for 24 h, while HepG2 cells were incubated for 8 h. RNA was extracted from the cells with the RNeasy Lipid Tissue Mini Kit and the RNeasy Mini Kit (Qiagen, Hilden, Germany), and cDNA was prepared from the RNA using ReverTra Ace® Quantitative reverse transcriptase PCR (qPCR) RT Master Mix with gDNA Remover (TOYOBO, Osaka, Japan). The qPCR was performed in duplicate using the MJ Mini instrument (Bio-Rad, Philadelphia, PA, USA) with the FastStart Universal SYBR Green Master mix (Roche Diagnostics, Indianapolis, IN). The cycling program used for PCR conditions was one cycle (one repeat with step 1, 50°C for 2 min and step 2, 95°C for 10 min), two cycles (39 repeats with step 1, 95°C for 15 s, and step 2, 60°C for 1 min) and three cycles (one repeat with step 1, 95°C for 10 s; step 2, 65°C for 5 s; and step 3, 95°C for 50 s). Glyceraldehyde-3-phosphate dehydrogenase (*Gapdh*) was used as a reference gene to normalize gene expression. All PCR primers were designed using the PrimerQuest Tool (Integrated DNA Technologies, purchased from Hokkaido System Science, Hokkaido, Japan). The sequences of the primers are presented in Supplementary Table S2. The qPCR data are shown as a percentage of the *Gapdh* mRNA expression level that was calculated as 100 × 1/2 (Ct of target gene−Ct of Gapdh gene) *(**33**)*. Reduction rates of mRNA expression by BU presented in Fig. 5H–K were calculated as Irr vs. KD of [% of *Gapdh* {(LPS –LPS + BU)/LPS}].

### Assessment of viability

Viability of BV2 cells treated with or without LPS and BU for 24 h was evaluated with the Alamar Blue (Eugene, OR, USA) assay as has been described previously *(**32**)*.

### Measurement of GSH

BV2 ells were incubated with or without LPS and BU for 4.5 h. Cellular GSH was measured by GSH measurement kit (DOJINDO Molecular Technologies, Tokyo, Japan) based on the GSH-reductase mediated enzyme recycling method together with the Ellman's reagent, DTNB [5,5′-dithiobis (2-nitrobenzoic acid)], according to the manufacturer's instruction. Absorbance of 415 nm light for produced 5-mercapto-2-nitrobenzoic acid was measured on EMax-Plus microplate reader controlled by SoftMaxPro software (Molecular Device).

### Chromatin immunoprecipitation assay

Chromatin Immunoprecipitation (ChIP) samples from BV2 cells treated with or without LPS and BU for 24 h were prepared with DNA purification buffers and spin columns (ChIP, cut & run) kit (Cell Signaling Technology), according to the manufacturer's instruction. Genomic DNA fragment immunoprecipitated by anti-NRF2 antibody (clone EP1808Y, rabbit monoclonal: Abcam, Cambridge, UK) or Normal Rabbit IgG antibody (Cell Signaling Technology) were subjected to PCR reactions to identify and measure relative amounts of precipitated DNA regions. Primer pairs used for detecting promoter regions upstream of transcription start sites were, mouse *Nqo1*: 5′-TGTATACCCAGGGAGCAGTT-3′ (sense), 5′-GAAGTCACCTTTGCACGCTA-3′ (antisense); and mouse *Rpl30* intron2 as negative control (Cell Signaling Technology).

### Measurement of nitrite

Conditioned media were obtained from BV2 cell cultures that had been incubated in the presence or absence of LPS and various reagents for 24 h, and NO in the media was determined based on the Griess reaction *(**32**)*. Only bicuculline was added to the culture 1 h before LPS and other agents were added. To normalize the released NO level by the cellular protein contents, the cells were solubilized with RIPA buffer, and the protein contents were determined using Pierce BCA protein assay reagents (Thermo Fisher Scientific, Rockford, IL).

**Fig. 4:**
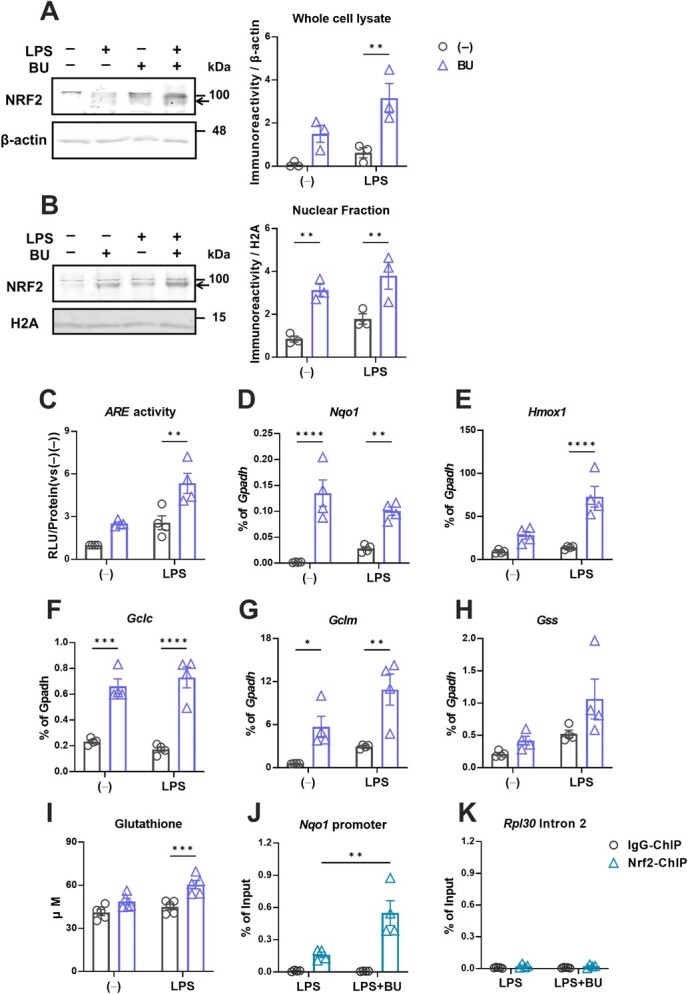
**BU enhances NRF2 stabilization and transcriptional activity in the murine microglial cell line, BV2*.*** Immunoblotting analysis and quantification of NRF2 protein (arrows) levels under BU and LPS stimulation in entire cell lysates (A) and nuclear extracts (B) of BV2 cells. Quantifications were executed using β-actin and histone 2A (H2A) blots as loading controls for whole cell lysates and nuclear extracts, respectively. Effects of BU on anti-oxidative NRF2 activity during LPS-mediated proinflammatory reactions in BV2 cells were examined and ARE activity measured by the luciferase reporter enhancer assay (C); mRNA levels of *Nqo1* (D); *Hmox1* (E); *Gclc* (F); *Gclm* (G); *Gss* (H); cellular levels of GSH (I); and binding of NRF2 to the ARE motif in the *Nqo1* promoter(J); Rpl30 Intron 2 (negative locus) (K) measured by the ChIP-qPCR assay, as described in **Methods**. The mRNA levels were represented as ratios of *Gapdh,* as shown in Fig. 2. The black circle plot in the quantification represents BU (−) and the blue triangle plot, BU (+). ^*^P < 0.05, ^**^P < 0.01 and ^***^P < 0.001 vs. BU (−).

### Statistical analysis

Data expressed as mean ± SD were statistically analysed using Prism 9 (GraphPad Software, La Jolla, CA). Data were subjected to two-tailed Student's *t* tests (unpaired) or two-way analysis of variance (ANOVA) followed by Šídák's multiple comparisons test. Significance was set at *P* < 0.05 unless otherwise stated.

**Fig. 5:**
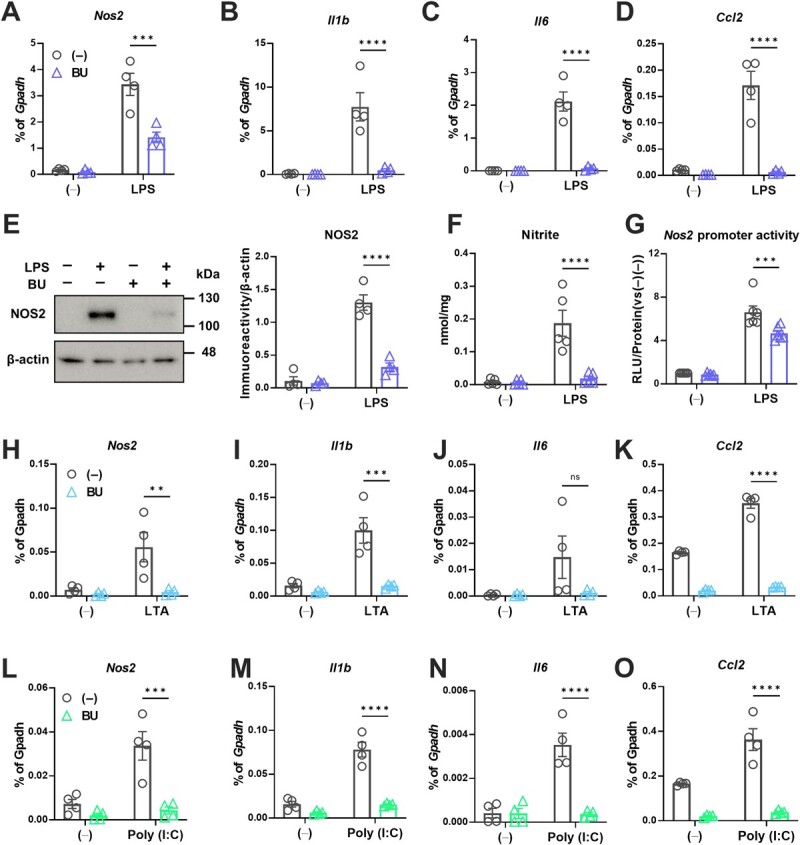
**BU acts as an anti-inflammatory agent by inhibiting the transcription of pro-inflammatory genes*.*** A–O. Effects of BU on LPS-mediated pro-inflammatory reaction in BV2 cells. mRNA expression of *Nos2* (A); *Il1-β* (B); *Il-6* (C); *Ccl2* (D); protein levels of NOS2 (E); cellular levels of nitrite (F); promoter activities of *Nos2* measured by the luciferase reporter enhancer assay (G), as described in **Methods.** β-actin blot was utilized as loading control for the quantification of NOS2 protein levels in E. H–K. Effects of BU on the mRNA levels of the pro-inflammatory genes under the LTA-mediated pro-inflammatory reaction (H, *Nos2*; I, *Il1b*; J, *Il6*; K *Ccl2*). L–O. Effects of BU on the mRNA levels of the pro-inflammatory genes under the Poly (I:C)-mediated pro-inflammatory reaction (L, *Nos2*; M, *Il1b*; N, *Il6*; O *Ccl2*). ^**^P < 0.01, ^***^P < 0.001, and ^****^P < 0.0001 vs. BU (−).

## Results

### BU dose-dependently enhanced ARE-dependent transcriptional activity

Using the human hepatoblastoma cell line HepG2, we examined whether BU affected the Keap1–Nrf2 pathway. The response element-dependent transcription and cell viability were simultaneously monitored using red-emitting (SLR3) and green-emitting (ELuc) beetle luciferases, respectively, through real-time bioluminescence measurement. We tested a concentration range of 6.25 μg/ml (28 μM) to 100 μg/ml (448 μM) because BU has been shown to exert anti-inflammatory effects without cytotoxicity at these doses in microglial cultures *(**32**)*. Figure 2A shows that BU dose-dependently activated ARE-dependent transcription (upper panels) without showing significant cytotoxicity at concentrations below 50 μg/ml, while some cytotoxicity was observed at 100 μg/ml (lower panels). An approximately 2-fold activation, which lasted for more than 60 h, was recorded at a concentration as low as 6.25 μg/ml, while the activation peaked at more than 8-fold and lasted longer than 60 h when 100 μg/ml of BU was used. To investigate the specificity of BU, we measured other stress response pathways, including NF-kB, ER stress, and heat shock response by monitoring NRE, ERSE and HSE-dependent transcriptional activity. Intriguingly, BU did not activate other stress response pathways but rather tended to suppress them even at 100 μg/ml dose (Fig. 2B–D). These results suggest that BU specifically activates the Keap1–Nrf2 pathway without another stress response pathway and that the Keap1–Nrf2 pathway is the primary target of BU.

### BU increased NRF2 protein levels and downstream gene expression in HepG2 cells

We examined NRF2 levels in HepG2 cells after eliminating KEAP1 by RNA interference to confirm whether KEAP1 was the target of BU activity. We found that NRF2 increased following *Keap1* knockdown with no noticeable additive effects after administering BU, suggesting that BU prevents the function of KEAP1 (Fig. 3A). Additionally, we evaluated the gene expression of the factors downstream of NRF2, besides *Nfe2l2* and *Keap1* themselves, in the *Keap1* knockdown cells (Fig. 3B–F). The mRNA level of *Nfe2l2* decreased after *Keap1* knockdown but was not affected further by BU (Fig. 3B), suggesting that the increase in NRF2 protein levels was via post-transcriptional regulation. The *Keap1* knockdown was confirmed while BU treatment increased *Keap1* mRNA levels in the siControl-treated cells (Fig. 3C). The downstream factors *Gclc*, *Gclm* and *Txnrd1* (Fig. 3D–F) were upregulated by BU in the siControl-treated cells and they remained unaltered in the absence of KEAP1, further confirming that BU upregulates NRF2 by targeting KEAP1.

**Fig. 6:**
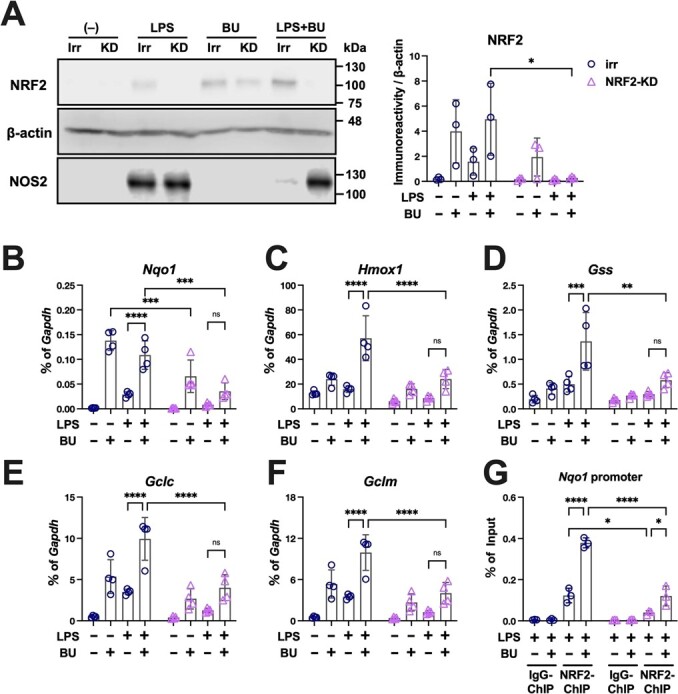
**Anti-oxidative effects of BU require NRF2.** A. Irr: BV2 cell stably expressing irrelevant sequence shRNA, and KD: BV2 cell expressing shRNA against NRF2. The reactivity to LPS and BU was assessed by measuring the expression of NRF2 and NOS2 proteins using immunoblot analyses with β-actin reblotting as the loading control. Quantification of the NRF2 signal normalized by β-actin is shown to the right of the immunoblot images. B–F. The mRNA levels of the downstream factors of NRF2 (B, *Nqo1*; C, *Hmox1*; D, *Gclc*; E *Gclm* and F, *Gss*), measured by qPCR, during LPS stimulation in the absence of NRF2, are represented as ratios of *Gapdh* as shown in Fig. 3. NRF2 binding to the ARE motif in the *Nqo1* promoter during LPS stimulation in the absence of NRF2 was examined by ChIP-qPCR (G). ^*^P < 0.05, ^**^P < 0.01, ^***^P < 0.001 and ^****^P < 0.0001 between the indicated conditions.

**Fig. 7:**
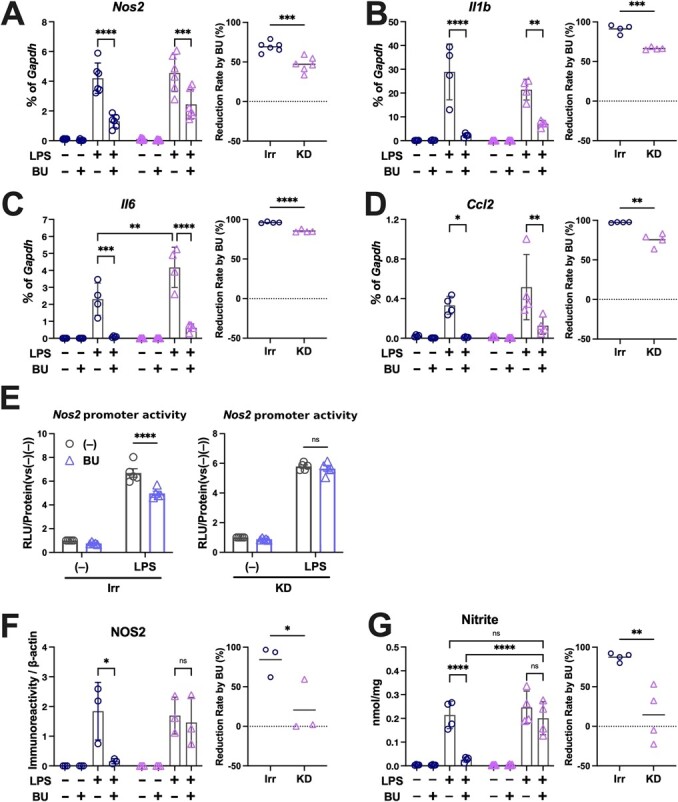
**Anti-inflammatory effects of BU require NRF2.** The expression of pro-inflammatory genes was measured using qPCR (A, *Nos2*; B, *Il1-* β; C, *Il-6* and D, *Ccl2,* each left panel), and the rates of reduction accompanied by the knockdown of NRF2 upon LPS stimulation (irr vs. KD of [% of *Gapdh* {(LPS –LPS + BU)/LPS}]) are plotted in each right panel. The promoter activities of the *Nos2* gene (E), NOS2 protein expression, as shown by the quantification of the NOS2 immunoblotting signal normalized to β-actin in Fig. 6A (F), and nitrite production (G) during LPS stimulation in the absence of NRF2 were also assessed. ^*^P < 0.05, ^**^P < 0.01, ^***^P < 0.001 and ^****^P < 0.0001 between the indicated conditions.

### BU enhanced NRF2-mediated transcriptional activity in a microglial cell line

Next, we examined whether this NRF2 pathway activation is translational to microglial cells, for which we reported anti-inflammatory and anti-oxidative functions by BU *(**22**,**32**)*. Similar to its effect on HepG2 cells, 100 μg/ml of BU increased NRF2 protein level in the total cell lysate of the murine microglial cell line BV2 (Fig. 4A). Although this dose was weakly cytotoxic for HepG2 cells (Fig. 2B and C), it did not affect the viability of BV2 cells, both in the presence and absence of lipopolysaccharide (LPS) (Supplementary Fig. S5). BU also increased NRF2 protein levels in the nuclear fraction of BV2 cells, implying that NRF2 was translocated into the nucleus after KEAP1-CUL3 complex-mediated degradation was stalled (Fig. 4B). BV2 cells were transfected with ARE activity reporter vector, and the effect of BU on ARE activity was examined (Fig. 4C). BU induced ARE-mediated transcriptional activation, predominantly during the inflammatory response by LPS (Fig. 4C). It also enhanced the mRNA levels of downstream genes transcriptionally regulated by NRF2, including *Nqo1*, *Hmox1*, *Gclc*, *Gclm* and *Gss* (Fig. 4D–H). The production of glutathione (GSH), which is catalysed by GCLC, GCLM and GSS, also increased after the addition of BU, especially during the pro-inflammatory reaction (Fig. 4I). We verified BU-induced NRF2 recruitment to the ARE site in the *Nqo1* promoter region during the inflammatory response using the chromatin immunoprecipitation (ChIP) assay (Fig. 4J). We also confirmed the selectivity of the NRF2 antibody by examining the signal from the negative locus (Fig. 4K). These data suggested that BU stabilized NRF2, resulting in the accelerated transcriptional control of the downstream factors.

### BU exerts anti-inflammatory actions in a microglial cell line

BV2 cells were responsive to LPS, promoting the expression of various pro-inflammatory mediators. BU suppressed the expression of these pro-inflammatory genes, such as inducible nitric oxide (NO) synthase (*Nos2*), *Il-1β*, *Il-6*, and C-C motif chemokine 2 *(Ccl2*), in LPS-treated BV2 cells (Fig. 5A–D). Furthermore, BU suppressed the expression of the NOS2 protein, generation of NO, and *Nos2* promoter activity in LPS-treated BV2 cells transfected with a *Nos2* promoter activity reporter vector (Fig. 5E–G). To examine the potential generality of these anti-inflammatory effects, we examined the effects of BU on other TLR ligands. Similar to the effect of LPS, BU suppressed the mRNA expression of *Nos2*, *Il1b*, *Il6*, and *Ccl2* upon stimulation with LTA (Fig. 5H–K) and poly (I:C) (Fig. 5L–O) that are TLR2 and TLR3 ligands, respectively. Overall, BU suppressed pro-inflammatory reactions in microglial cells under various stimuli.

### Anti-inflammatory and anti-oxidative effects of BU requiring NRF2

BV2 cells expressing a small hairpin RNA targeting NRF2 (KD) or an irrelevant sequence (Irr) were established (Fig. 6). NRF2 protein was not detected in the KD cells in the absence of BU (Fig. 6A). LPS-induced NOS2 and NRF2 expression in Irr cells and BU strongly suppressed the former while elevating the latter in these cells. However, KD cells strongly expressed NOS2 even in the presence of BU with the absence of upregulation of NRF2 (Figs. 6A and 7F).

BU increased the expression of antioxidant genes in LPS-treated Irr cells, but its effects were strongly attenuated in KD cells (Fig. 6B–F). The ChIP assay revealed that NRF2 recruitment to the ARE motif in the *Nqo1* promoter was also remarkably suppressed in the KD cells (Fig. 6G), highlighting the significance of the NRF2 protein in transcriptional control. BU markedly increased NRF2 recruitment to the *Nqo1* promoter in Irr cells.

Conversely, BU suppressed the LPS-induced expression of pro-inflammatory genes, like *Nos2*, *Il-1β*, *Il-6* and *Ccl2*, both in Irr and KD cells (Fig. 7A–D). However, its anti-inflammatory effects were significantly diminished in the KD cells, as shown in the graphs comparing reduction rates by BU (Fig. 7A–D, right panels). The inhibitory effect of BU on the *Nos2* promoter was lost in the KD cells (Fig. 7E), in accordance with the BU-mediated suppression of NOS2 protein expression (Figs. 6A and 7F) and nitrite production (Fig. 7G). Collectively, these data emphasize the participation of NRF2 in the anti-inflammatory and anti-oxidative activities of BU.

## Discussion

In this study, we found that BU significantly enhances the accumulation of NRF2 and the expression of downstream target genes. Furthermore, we also confirmed the participation of NRF2 in the anti-inflammatory property of BU using murine microglial cell line, BV2.

NRF2 is a fundamental regulator of oxidative stress and inflammation. Reciprocal interactions between oxidative damage and inflammation synergistically exacerbate the pathophysiology of many diseases *(**34**,**35**)*. Microglia, the brain-resident immune cells, play a central role in various central nervous system diseases, including ischemia, traumatic injuries, degeneration, and tumors, all of which accompany neuroinflammation. In such pathological conditions, activated microglia are entrenched in a vicious cycle involving the release of potentially neurotoxic substances, such as reactive oxygen species (ROS), pro-inflammatory cytokines, and glutamate *(**36**,**37**)*. Therefore, microglial cells have been investigated as drug targets to reduce the release of these neurotoxic factors *(**38**)*. NRF2 exerts anti-oxidative and anti-inflammatory effects by binding to ARE and transcriptionally activating downstream anti-oxidative genes *(**39**)*. Genetic and pharmacological activation of NRF2 has protective effects in various disease models involving inflammation and oxidative stress *(**40**,**41**)*. Hence, various NRF2 activators have been investigated, including DMF, which has been clinically approved *(**42**,**43**)*, and a few compounds are currently under phase II/III clinical trials *(**13**)*.

In this study, we revealed for the first time that BU boosts NRF2 activity by increasing its levels and translocation into the nucleus. Furthermore, the enhancement of ARE-mediated transcriptional activity by BU lasted over 60 h. An NRF2 activator, 5-hydroxy-4-phenyl-butenolide, has been shown to exert anti-oxidative action by activating NRF2, but the action peaked 5 h after addition and rapidly disappeared *(**27**)*–very different from the persistent action of BU on ARE-dependent transcription. Although we do not know the mechanism behind this, plausible explanations include the stability inside the medium/cells, such as resistance to degradation, and any other mechanism to produce degraded products or endogenous metabolites that could inhibit KEAP1. Another explanation is related to the nuclear export system. NRF2 is exported after nuclear translocation and is regulated by phosphorylation at Y568. The exported NRF2 is then subjected to degradation *(**44**)*. Hence, it is possible that BU inhibits the phosphorylation of NRF2, leading to longer nuclear residence. These mechanisms, which are unique to BU, possibly participate in the long-lasting kinetics.

BU increased NRF2 protein levels, which was consistent with the *Keap1* KD conditions. It has now been established that NRF2 escapes KEAP1-mediated degradation when the cysteine residues of KEAP1 are modified by electrophiles *(**5**)*. α-Bromo carbonyl compounds, including BU, have highly electrophilic structures, which contain the lowest unoccupied molecular orbital (LUMO) formed by two neighboring powerfully electrophilic carbon atoms *(**45**)*. Hence, we speculate that BU can cause electrophilic reactions with cysteine residues, such as KEAP1, leading to the stabilization of NRF2. Further studies are warranted to elucidate these target cysteine residues.

In microglial cell line BV2, the inhibitory effects of BU on LPS-induced *Nos2* and other pro-inflammatory gene expression were significantly attenuated in the *Nrf2*-KD cells; however, the reduction was not robust enough to nullify the overall effect of BU. Simultaneously, the inhibitory effect of BU on the promoter activity of *Nos2* (approximately 30% reduction, Fig. 5L left panel) was lost in *Nrf2*-KD cells (Fig. 5L right panel), suggesting that NRF2 participates, at least partly, in BU's transcriptional suppression of pro-inflammatory genes. Intriguingly, the BU-induced suppression of NOS2 protein expression was severely diminished in KD cells (Fig. 6A and Fig. 7F), implying that post-transcriptional control of NOS2 expression by NRF2 plays a major role in the overall suppression of NO production by BU. Hence, the anti-inflammatory properties of BU, particularly in terms of transcriptional control, in which the participation of NRF2 is not fully elucidated *(**12**)*, do not entirely depend on NRF2, and combined effects with other anti-inflammatory mechanisms, such as Janus kinase 1 inhibition *(**32**)* and/or reduction of cellular energy metabolism *(**22**)*, might be involved.

Due to BU-induced elevated NRF2 activity, the expression of anti-oxidative genes (*Nqo1*, *Gclc*, *Gclm*, *Gss*) and the GSH content increased. These changes should elevate cellular antioxidant capacity. In fact, BU administration in a traumatic brain injury (TBI) rat model reduced ROS-induced cellular injuries, as revealed by the reduction of 8-hydroxy-2′-deoxyguanosine in neurons *(**22**)*. BU treatment was effective in TBI or Parkinson's disease rat models, wherein inflammatory and oxidative reactions by immune cells, including microglia, play critical aggravating roles *(**21**,**22**)*.

This study suggests that BU could be repurposed as a novel, clinically approved NRF2 activator. Although we do not provide any *in vivo* evidence for NRF2 activation, previous reports demonstrated that BU, at a dose of 33–50 mg/kg/day exerts an anti-inflammatory effect in several rat disease models *(**20*–*22**)*. On the other hand, according to the package insert of medication containing BU, such as BROVARIN^®^ (Nihon Shinyaku, Kyoto, Japan) and Naronace^®^ (Taisho Pharmaceutical, Tokyo, Japan), the maximum oral intoxication dosage is 600 to 800 mg/day (10–13 mg/kg/day). Given that rodents have faster metabolic rates than humans and that human equivalent doses can be calculated by dividing the rat dose by 6.2 *(**46**)*, the doses that showed anti-inflammatory effects in rat models can be clinically translational. Hence, we speculate that both the anti-inflammatory and anti-oxidative effects mediated by the activation of NRF2 could potentially be translated to humans.

Repurposing ‘old’ drugs to treat both common and rare diseases is becoming increasingly attractive because it involves using de-risked compounds, potentially lowering overall development costs and shortening development timelines *(**47**)*. Because BU has been prescribed to patients since the early 1900s *(**16**)*, its clinical safety may be established when it is used as an anti-inflammatory drug. However, the major limitation of our study is that we did not provide in vivo evidence for NRF2 activation. Hence, further studies need to be conducted to confirm whether BU's beneficial effects in various disease models involve the activation of NRF2.

## Supplementary Material

Web_Material_mvad030

## References

[1] Nathan, C. and Ding, A. (2010) Nonresolving inflammation. Cell 140, 871–882. doi: 10.1016/j.cell.2010.02.029 20303877

[2] Sokolove, J. and Lepus, C.M. (2013) Role of inflammation in the pathogenesis of osteoarthritis: latest findings and interpretations. Ther Adv Musculoskelet Dis 5, 77–94. doi: 10.1177/1759720X12467868 23641259 PMC3638313

[3] Siti, H.N., Kamisah, Y., and Kamsiah, J. (2015) The role of oxidative stress, antioxidants and vascular inflammation in cardiovascular disease (a review). Vasc. Pharmacol. 71, 40–56. doi: 10.1016/j.vph.2015.03.005 25869516

[4] Saha, S., Buttari, B., Profumo, E., Tucci, P., and Saso, L. (2021) A perspective on Nrf2 Signaling pathway for Neuroinflammation: a potential therapeutic target in Alzheimer’s and Parkinson’s diseases. Front. Cell. Neurosci. 15, 1–15. doi: 10.3389/fncel.2021.787258 PMC8813964 35126058

[5] Yamamoto, M., Kensler, T.W., and Motohashi, H. (2018) The KEAP1-NRF2 system: a thiol-based sensor-effector apparatus for maintaining redox homeostasis. Physiol. Rev. 98, 1169–1203. doi: 10.1152/physrev.00023.2017 29717933 PMC9762786

[6] Matsumaru, D. and Motohashi, H. (2021) The KEAP1-NRF2 system in healthy aging and longevity. Antioxidants 10, 1929–1948. doi: 10.3390/antiox10121929 34943032 PMC8750203

[7] Saito, R., Suzuki, T., Hiramoto, K., Asami, S., Naganuma, E., Suda, H., Iso, T., Yamamoto, H., Morita, M., Baird, L., Furusawa, Y., Negishi, T., Ichinose, M., and Yamamoto, M. (2016) Characterizations of three major cysteine sensors of Keap1 in stress response. Mol. Cell. Biol. 36, 271–284. doi: 10.1128/mcb.00868-15 26527616 PMC4719294

[8] Suzuki, T. and Yamamoto, M. (2017) Stress-sensing mechanisms and the physiological roles of the Keap1–Nrf2 system during cellular stress. J. Biol. Chem. 292, 16817–16824. doi: 10.1074/jbc.R117.800169 28842501 PMC5641889

[9] Zhang, J., Ohta, T., Maruyama, A., Hosoya, T., Nishikawa, K., Maher, J.M., Shibahara, S., Itoh, K., and Yamamoto, M. (2006) BRG1 interacts with Nrf2 to selectively mediate HO-1 induction in response to oxidative stress. Mol. Cell. Biol. 26, 7942–7952. doi: 10.1128/mcb.00700-06 16923960 PMC1636732

[10] Katsuoka, F. and Yamamoto, M. (2016) Small Maf proteins (MafF, MafG, MafK): history, structure and function. Gene 586, 197–205. doi: 10.1016/j.gene.2016.03.058 27058431 PMC4911266

[11] Sekine, H., Okazaki, K., Ota, N., Shima, H., Katoh, Y., Suzuki, N., Igarashi, K., Ito, M., Motohashi, H., and Yamamoto, M. (2016) The mediator subunit MED16 transduces NRF2-activating signals into antioxidant gene expression. Mol. Cell. Biol. 36, 407–420. doi: 10.1128/MCB.00785-15 26572828 PMC4719425

[12] Kobayashi, E.H., Suzuki, T., Funayama, R., Nagashima, T., Hayashi, M., Sekine, H., Tanaka, N., Moriguchi, T., Motohashi, H., Nakayama, K., and Yamamoto, M. (2016) Nrf2 suppresses macrophage inflammatory response by blocking proinflammatory cytokine transcription. Nat. Commun. 7, 1–14. doi: 10.1038/ncomms11624 PMC4879264 27211851

[13] Robledinos-Antón, N., Fernández-Ginés, R., Manda, G., and Cuadrado, A. (2019) Activators and inhibitors of NRF2: a review of their potential for clinical development. Oxidative Med. Cell. Longev. 2019, 1–20. doi: 10.1155/2019/9372182 PMC6664516 31396308

[14] Cuadrado, A., Pajares, M., Benito, C., Jiménez-Villegas, J., Escoll, M., Fernández-Ginés, R., Garcia Yagüe, A.J., Lastra, D., Manda, G., Rojo, A.I., and Dinkova-Kostova, A.T. (2020) Can activation of NRF2 be a strategy against COVID-19? Trends Pharmacol. Sci. 41, 598–610. doi: 10.1016/j.tips.2020.07.003 32711925 PMC7359808

[15] Jordan, A.L.M., Yang, J., Fisher, C.J., Racke, M.K., and Mao-Draayer, Y. (2022) Progressive multifocal leukoencephalopathy in dimethyl fumarate-treated multiple sclerosis patients. Mult. Scler. J. 28, 7–15. doi: 10.1177/1352458520949158 PMC7889744 32808554

[16] Eeckhout, A. (1907) Studien über die hypnotische Wirkung in der Vaïerian-süuregruppe. Arch.Exp.Path.und Pharmak. 57, 338–357

[17] Yan, M.T., Yang, S.S., Chu, H.Y., and Lin, S.H. (2009) Ataxia associated with spurious hyperchloremia: the one behind the scene. Am. J. Emerg. Med. 27, 752.e1–752.e3. doi: 10.1016/j.ajem.2008.09.044 19751639

[18] Biyajima, M., Satoh, S., Morikawa, T., Morita, Y., Watanabe, R., Matsui, D., Konno, M., Morimoto, N., Yatsu, Y., Hirasaki, A., and Yahikozawa, H. (2022) Bromisoval-induced bromism with status epilepticus mimicking Wernicke’s encephalopathy: report of two cases. BMC Neurol. 22, 181–185. doi: 10.1186/s12883-022-02712-3 35578314 PMC9109394

[19] Yanagisawa, K. (2015) Transition of psychotropic drugs in Japanese pharmacopoeia (JP) (part 16). Transitions in the standards and test methods of bromovalerylurea in JP V (1932) and JP X VI (2011), and comparison with Deutsches Arzneibuch. Yakushigaku Zassi 50, 143–158 27149780

[20] Kikuchi, S., Nishihara, T., Kawasaki, S., Abe, N., Kuwabara, J., Choudhury, M.E., Takahashi, H., Yano, H., Nagaro, T., Watanabe, Y., Aibiki, M., and Tanaka, J. (2015) The ameliorative effects of a hypnotic bromvalerylurea in sepsis. Biochem. Biophys. Res. Commun. 459, 319–326. doi: 10.1016/j.bbrc.2015.02.111 25732089

[21] Higaki, H., Choudhury, M.E., Kawamoto, C., Miyamoto, K., Islam, A., Ishii, Y., Miyanishi, K., Takeda, H., Seo, N., Sugimoto, K., Takahashi, H., Yano, H., and Tanaka, J. (2016) The hypnotic bromovalerylurea ameliorates 6-hydroxydopamine-induced dopaminergic neuron loss while suppressing expression of interferon regulatory factors by microglia. Neurochem. Int. 99, 158–168. doi: 10.1016/j.neuint.2016.06.013 27392596

[22] Abe, N., Choudhury, M.E., Watanabe, M., Kawasaki, S., Nishihara, T., Yano, H., Matsumoto, S., Kunieda, T., Kumon, Y., Yorozuya, T., and Tanaka, J. (2018) Comparison of the detrimental features of microglia and infiltrated macrophages in traumatic brain injury: a study using a hypnotic bromovalerylurea. Glia 66, 2158–2173. doi: 10.1002/glia.23469 30194744

[23] Ishii, Y., Yamaizumi, A., Kawakami, A., Islam, A., Choudhury, M.E., Takahashi, H., Yano, H., and Tanaka, J. (2015) Anti-inflammatory effects of noradrenaline on LPS-treated microglial cells: suppression of NFκB nuclear translocation and subsequent STAT1 phosphorylation. Neurochem. Int. 90, 56–66. doi: 10.1016/j.neuint.2015.07.010 26190182

[24] Nakajima, Y., Yamazaki, T., Nishii, S., Noguchi, T., Hoshino, H., Viviani, V.R., and Ohmiya, Y. (2010) Enhanced beetle luciferase for high-resolution bioluminescence imaging. PLoS One 5, e10011–e10022. doi: 10.1371/journal.pone.0010011 20368807 PMC2848861

[25] Viviani, V.R., Bechara, E.J.H., and Ohmiya, Y. (1999) Cloning, sequence analysis, and expression of active Phrixothrix railroad-worms luciferases: relationship between bioluminescence spectra and primary structures. Biochemistry 38, 8271–8279. doi: 10.1021/bi9900830 10387072

[26] Uno, K., Murotomi, K., Kazuki, Y., Oshimura, M., and Nakajima, Y. (2018) Bioluminescence-based cytotoxicity assay for simultaneous evaluation of cell viability and membrane damage in human hepatoma HepG2 cells. Luminescence 33, 616–624. doi: 10.1002/bio.3454 29424036

[27] Tabei, Y., Murotomi, K., Umeno, A., Horie, M., Tsujino, Y., Masutani, B., Yoshida, Y., and Nakajima, Y. (2017) Antioxidant properties of 5-hydroxy-4-phenyl-butenolide via activation of Nrf2/ARE signaling pathway. Food Chem. Toxicol. 107, 129–137. doi: 10.1016/j.fct.2017.06.039 28655653

[28] Wakuri, S., Yamakage, K., Kazuki, Y., Kazuki, K., Oshimura, M., Aburatani, S., Yasunaga, M., and Nakajima, Y. (2017) Correlation between luminescence intensity and cytotoxicity in cell-based cytotoxicity assay using luciferase. Anal. Biochem. 522, 18–29. doi: 10.1016/j.ab.2017.01.015 28111305

[29] Nakajima, Y., Kimura, T., Sugata, K., Asakawa, A., Kubota, H., Ikeda, M., and Ohmiya, Y. (2005) Multicolor luciferase assay system: one-step monitoring of multiple gene expressions with a single substrate. BioTechniques 38, 891–894 16018550 10.2144/05386ST03

[30] Noguchi, T., Michihata, T., Nakamura, W., Takumi, T., Shimizu, R., Yamamoto, M., Ikeda, M., Ohmiya, Y., and Nakajima, Y. (2010) Dual-color luciferase mouse directly demonstrates coupled expression of two clock genes. Biochemistry 49, 8053–8061. doi: 10.1021/bi100545h 20718447

[31] Kaminota, T., Yano, H., Shiota, K., Nomura, N., Yaguchi, H., Kirino, Y., Ohara, K., Tetsumura, I., Sanada, T., Ugumori, T., Tanaka, J., and Hato, N. (2017) Elevated Na+/H+ exchanger-1 expression enhances the metastatic collective migration of head and neck squamous cell carcinoma cells. Biochem. Biophys. Res. Commun. 486, 101–107. doi: 10.1016/j.bbrc.2017.03.007 28268168

[32] Kawasaki, S., Abe, N., Ohtake, F., Islam, A., Choudhury, M.E., Utsunomiya, R., Kikuchi, S., Nishihara, T., Kuwabara, J., Yano, H., Watanabe, Y., Aibiki, M., Yorozuya, T., and Tanaka, J. (2017) Effects of hypnotic bromovalerylurea on microglial BV2 cells. J. Pharmacol. Sci. 134, 116–123. doi: 10.1016/j.jphs.2017.05.007 28645489

[33] Islam, A., Choudhury, M.E., Kigami, Y., Utsunomiya, R., Matsumoto, S., Watanabe, H., Kumon, Y., Kunieda, T., Yano, H., and Tanaka, J. (2018) Sustained anti-inflammatory effects of TGF-β1 on microglia/macrophages. Biochim. Biophys. Acta Mol. basis Dis. 1864, 721–734. doi: 10.1016/j.bbadis.2017.12.022 29269050

[34] Barnham, K.J., Masters, C.L., and Bush, A.I. (2004) Neurodegenerative diseases and oxidatives stress. Nat. Rev. Drug Discov. 3, 205–214. doi: 10.1038/nrd1330 15031734

[35] Ferrucci, L. and Fabbri, E. (2018) Inflammageing: chronic inflammation in ageing, cardiovascular disease, and frailty. Nat. Rev. Cardiol. 15, 505–522. doi: 10.1038/s41569-018-0064-2 30065258 PMC6146930

[36] Takeuchi, H., Jin, S., Suzuki, H., Doi, Y., Liang, J., Kawanokuchi, J., Mizuno, T., Sawada, M., and Suzumura, A. (2008) Blockade of microglial glutamate release protects against ischemic brain injury. Exp. Neurol. 214, 144–146. doi: 10.1016/j.expneurol.2008.08.001 18775425

[37] Patel, A.R., Ritzel, R., McCullough, L.D., and Liu, F. (2013) Microglia and ischemic stroke: a double-edged sword. Int J Physiol Pathophysiol Pharmacol 5, 73–90 23750306 PMC3669736

[38] Takeda, H., Yamaguchi, T., Yano, H., and Tanaka, J. (2021) Microglial metabolic disturbances and neuroinflammation in cerebral infarction. J. Pharmacol. Sci. 145, 130–139. doi: 10.1016/j.jphs.2020.11.007 33357771

[39] Ahmed, S.M.U., Luo, L., Namani, A., Wang, X.J., and Tang, X. (2017) Nrf2 signaling pathway: pivotal roles in inflammation. Biochim. Biophys. Acta Mol. basis Dis. 1863, 585–597. doi: 10.1016/j.bbadis.2016.11.005 27825853

[40] Zhao, M., Murakami, S., Matsumaru, D., Kawauchi, T., Nabeshima, Y., and Motohashi, H. (2022) NRF2 pathway activation attenuates ageing-related renal phenotypes due to α-klotho deficiency. The Journal of Biochemistry 171, 579–589. doi: 10.1093/jb/mvac014 35137128

[41] Duan, C., Wang, H., Jiao, D., Geng, Y., Wu, Q., Yan, H., and Li, C. (2022) Curcumin restrains oxidative stress of after Intracerebral Hemorrhage in rat by activating the Nrf2/HO-1 pathway. Front. Pharmacol. 13, 1–15. doi: 10.3389/fphar.2022.889226 PMC9092178 35571134

[42] Burness, C.B. and Deeks, E.D. (2014) Dimethyl fumarate: a review of its use in patients with relapsing-remitting multiple sclerosis. CNS Drugs 28, 373–387. doi: 10.1007/s40263-014-0155-5 24623127

[43] Majkutewicz, I. (2022) Dimethyl fumarate : a review of preclinical efficacy in models of neurodegenerative diseases. Eur. J. Pharmacol. 926,175025–175040. doi: 10.1016/j.ejphar.2022.175025 35569547

[44] Niture, S.K., Khatri, R., and Jaiswal, A.K. (2014) Regulation of Nrf2 - an update. Free Radic. Biol. Med. 66, 36–44. doi: 10.1016/j.freeradbiomed.2013.02.008 23434765 PMC3773280

[45] Clayden, J. and Nick Greeves, S.W. (2012) ORGANIC CHEMISTRY, 2nd edn. pp 341–342 Oxford University Press, the U.K, Great Clarendon Street, Oxford, OX2 6DP, the U.K

[46] Nair, A. and Jacob, S. (2016) A simple practice guide for dose conversion between animals and human. J Basic Clin Pharm 7, 27–31. doi: 10.4103/0976-0105.177703 27057123 PMC4804402

[47] Pushpakom, S., Iorio, F., Eyers, P.A., Escott, K.J., Hopper, S., Wells, A., Doig, A., Guilliams, T., Latimer, J., McNamee, C., Norris, A., Sanseau, P., Cavalla, D., and Pirmohamed, M. (2019) Drug repurposing: Progress, challenges and recommendations. Nat. Rev. Drug Discov. 18, 41–58. doi: 10.1038/nrd.2018.168 30310233

