# Supplementary material for: The anti-inflammatory and anti-oxidative effect of a classical hypnotic bromovalerylurea mediated by the activation of NRF2
Source: J Biochem. 2023 Apr 11;174(2):131–42. doi: 10.1093/jb/mvad030 (PMC10372716; doi:10.1093/jb/mvad030)
Supplement: Web_Material_mvad030 [file web_material_mvad030.zip › 230310_Takeda_et_al_Ehime_Univ_Supplementary_Materials.docx]

**The anti-inflammatory and anti-oxidative effect of a classical hypnotic bromovalerylurea mediated by the activation of NRF2**

Haruna Takeda^a,c^, Yoshihiro Nakajima^b^, Teruaki Yamaguchi^a^, Hiroki Sekine^c^, Hozumi Motohashi ^c^, Hajime Yano^a*^, Junya Tanaka^a^

Running title: Activation of NRF2 by bromovalerylurea

^a^ Department of Molecular and Cellular Physiology, Graduate School of Medicine, Ehime University, Toon, Ehime, Japan

^b^ Health and Medical Research Institute, National Institute of Advanced Industrial Science and Technology (AIST), Takamatsu, Kagawa, Japan

^c^ Department of Gene Expression Regulation, Institute of Development, Aging and Cancer, Tohoku University, Sendai, 980-8575, Japan

* Correspondence to: Hajime Yano, Department of Molecular and Cellular Physiology, Graduate School of Medicine, Ehime University, Toon, Ehime 791-0295, Japan; E-mail addresses: [hajime-y@m.ehime-u.ac.jp](mailto:jtanaka@m.ehime-u.ac.jp)

**Supplementary Table 1. Antibodies**

| **Name** | **Host** | **Cat** |
| --- | --- | --- |
| NRF2 (mouse) I.B. | Rabbit polyclonal | Proteintech, Cat# 16396-1-AP |
| NRF2 (mouse) (ChIP) | Rabbit monoclonal  Clone# EP1808Y | abcam, Cat#ab62352 |
| β-actin | Mouse monoclonal  Clone# AC-15 | Sigma-aldrich, Cat#A1978 |
| H2A | Rabbit polyclonal | Proteintech, Cat#10856-1-AP |
| NRF2 (human) | Rabbit polyclonal | Santa Cruz, sc-13032 |
| Lamin B1 | Rabbit polyclonal | MBL, PM064 |

**Supplementary Table 2. Primers used for qPCR**

| Name | Sequence (5’–3’) |
| --- | --- |
| Human *Gapdh* Forward | GAAGGTGAAGGTCGGAGTC |
| Human *Gapdh* Reverse | GAAGATGGTGATGGGATTTC |
| Human Nfe2l2 Forward | TCATGATGGACTTGGAGCTG |
| Human Nfe2l2 Reverse | CATACTCTTTCCGTCGCTGA |
| Human Keap1 Forward | CTGGAGGATCATACCAAGCAGG |
| Human Keap1 Reverse | GAACATGGCCTTGAAGACAGG |
| Human Gclc Forward | TCTCTAATAAAGAGATGAGCAACATGC |
| Human Gclc Reverse | TTGACGATAGATAAAGAGATCTACGAA |
| Human Gclm Forward | TAGAATCAAACTCTTCATCATCAACTAGA |
| Human Gclm Reverse | TCACAGAATCCAGCTGTGCAA |
| Human Txnrd1 Forward | GCATCCCTGGTGACAAAGAA |
| Human Txnrd1 Reverse | GCACTCCAAAGCGACATAGG |
| Mouse Gapdh Forward | ACCCAGAAGACTGTGGATGG |
| Mouse Gapdh Reverse | CACATTGGGGGTAGGAACAC |
| Mouse Nqo1 Forward | TTCTCTGGCCGATTCAGAGT |
| Mouse Nqo1 Reverse | GGCTGCTTGGAGCAAAATAG |
| Mouse Hmox1 Forward | TGAAGGAGGCCACCAAGGAGG |
| Mouse Hmox1 Reverse | AGAGGTCACCCAGGTAGCGGG |
| Mouse Gclc Forward | GATGTGGACACCCGATGCAG |
| Mouse Gclc Reverse | CTTGCTGTAGTCAGGATGG |
| Mouse Gclm Forward | TGCCACAGATTTGACTGCC |
| Mouse Gclm Reverse | TCAGGGATGCTTTCTTGAAG |
| Mouse Gss Forward | GCGGTGGTGCTACTGATTGC |
| Mouse Gss Reverse | CATCCATAAACAGCCTTCGG |
| Mouse Nos2 Forward | AACGGAGAACGTTGGATTTG |
| Mouse Nos2 Reverse | TTCTGTGCTGTCCCAGTGAG |
| Mouse Il1b Forward | GGGCCTCAAAGGAAAGAATC |
| Mouse Il1b Reverse | TACCAGTTGGGGAACTCTGC |
| Mouse Il6 Forward | CTGATGCTGGTGACAACCAC |
| Mouse Il6 Reverse | CAGAATTGCCATTGCACAAC |
| Mouse Ccl2 Forward | CATCCACGTGTTGGCTCA |
| Mouse Ccl2 Reverse | GATCATCTTGCTGGTGAATGAGT |

**Supplementary Figure S1. Changes in ARE-TK-SLR3 intensity at different concentrations of BU in real-time bioluminescence recordings.**

The time-dependent changes in ARE-mediated transcriptional activations where the bioluminescence intensity (SLR3) of the BU-treated cells were normalized to that of vehicle control cells (expressed as 100%) and presented as percentage of control at each time point. Dose-dependencies are summarized in the areas under the curves (AUC) at the right-most panel. ARE-dependent transcription, which was calculated as log_2_ (ARE-TK-SLR3 (% of Control) /TK-ELuc (% of Control)), is shown in Fig 2A (upper panels).

**Supplementary Figure S2. Changes in NRE-TK-SLR3 and TK-ELuc intensity at different concentrations of BU in real-time bioluminescence recordings.**

The time-dependent changes in NRE-mediated transcriptional activations where the bioluminescence intensity (SLR3) of the BU-treated cells were normalized to that of vehicle control cells (expressed as 100%) and presented as percentage of control at each time point (upper panels). Simultanously monitored viability (ELuc, same calculation as SLR3) was shown in lower panels. Dose-dependencies are summarized in the areas under the curves (AUC) at the right-most panel. NRE-dependent transcription, which was calculated as log_2_ (NRE-TK-SLR3 (% of Control) / TK-ELuc (% of Control)), is shown in Fig 2B.

**Supplementary Figure S3. Changes in ERSE-TK-SLR3 and TK-ELuc intensity at different concentrations of BU in real-time bioluminescence recordings.**

The time-dependent changes in ERSE-mediated transcriptional activations where the bioluminescence intensity (SLR3) of the BU-treated cells were normalized to that of vehicle control cells (expressed as 100%) and presented as percentage of control at each time point (upper panels). Simultanously monitored viability (ELuc, same calculation as SLR3) was shown in lower panels. Dose-dependencies are summarized in the areas under the curves (AUC) at the right-most panel. ERSE-dependent transcription, which was calculated as log_2_ (ERSE-TK-SLR3 (% of Control)/TK-ELuc (% of Control)), is shown in Fig 2C.

**Supplementary Figure S4. Changes in HSE-TK-SLR3 and TK-ELuc intensity at different concentrations of BU in real-time bioluminescence recordings.**

The time-dependent changes in HSE-mediated transcriptional activations where the bioluminescence intensity (SLR3) of the BU-treated cells were normalized to that of vehicle control cells (expressed as 100%) and presented as percentage of control at each time point (upper panels). Simultanously monitored viability (ELuc, same calculation as SLR3) was shown in lower panels. Dose-dependencies are summarized in the areas under the curves (AUC) at the right-most panel. HSE-dependent transcription, which was calculated as log_2_ (ERSE-TK-SLR3 (% of Control)/TK-ELuc (% of Control)), is shown in Fig 2D.


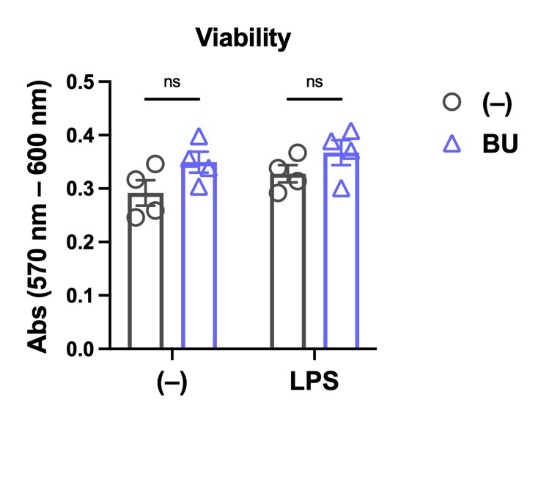


**Supplementary Figure S5. 100 μg/ml of BU had no significant effects on BV2 viability**

Cells were incubated with or without LPS (1 μg/ml) and BU (100 μg/ml) for 24 h. Subsequently, the cells were incubated with Alamar Blue, and the optical density changes in the conditioned media were measured. However, BU did not affect the viability.
